# Assessing the Needs of People with Disabilities for Physical Activities and Sports in South Korea

**DOI:** 10.3390/healthcare10020265

**Published:** 2022-01-29

**Authors:** Ahra Oh, Wi-Young So

**Affiliations:** 1 Department of Exercise Rehabilitation & Welfare, Gachon University, 191 Hombakmoero, Yeonsu-gu, Incheon 21936, Korea; dhdkfk@gachon.ac.kr; 2 Sport Medicine Major, College of Humanities and Arts, Korea National University of Transportation, Chungju-si 27469, Korea

**Keywords:** people with disabilities, physical activities, South Korea, social determinants of health, focus group interviews

## Abstract

This study’s objective was to understand the physical activities and sports-related needs of people with disabilities in South Korea and how those needs should be reflected in policy and practice to improve these people’s quality of life. Accordingly, focus group interviews were conducted with 35 people with disabilities who had participated in physical activities. Interviews were conducted one-on-one or in small groups of three or four. The interview questions related to their participation experiences in physical activities and sports, their difficulties with such participation, and their thoughts on what was needed to improve their participation. For data analysis, the interviews were transcribed and the content analyzed, with content triangulation performed for validity. From this, a total of 307 meaningful references were derived, comprising four categories, eight theme clusters, and 40 themes. The current physical activities and sports programs for people with disabilities in South Korea are led by the government to provide an environment for them to participate; however, to improve the quality of life for these people, these must be transformed into consumer-centric programs. To provide an opportunity for people with disabilities to choose the exercise program of their choice, it is necessary to research in advance what kind of exercise program these people want, centering on the local community. To strengthen the professionalism of disabled sports instructors, it is necessary to provide a stable environment for them in sports facilities and continue training them to build capacity. In terms of facilities, the expansion of sports facilities that can be used by people with disabilities is an urgent priority, and the opinions of users with disabilities must be actively collected and addressed in the operation of these facilities. Additionally, at the national level, information should be continuously provided through mass media and the Internet so that people with disabilities can know the importance of physical activity and sports and manage their own health. To that end, it would be helpful to conduct an in-depth analysis of countries with effective participation policies for people with disabilities and consider how these could be adapted to the situation in Korea.

## 1. Introduction

Since the 1988 Seoul Paralympic Games, there has been a growth in South Korea (henceforth “Korea”) of sports and physical activities for people with disabilities, with the full support of the Korean government [1]. Since then, the government launched has its first and now its third “Plan for the Promotion of Sports for People with Disabilities” covering 2008 to 2022, and continues to develop these plans [2].

In 2019, the Ministry of Culture, Sports and Tourism, which is in charge of sports administration in Korea, received a budget increase of 145% “to stimulate physical activities and sports” in the country [3]. In addition, this government agency is responsible for promoting projects such as the building of 150 sports facilities for people with disabilities by 2025, arranging for 1200 adapted instructors for the disabled by 2022, and introducing free sports lessons for the disabled [4]. The enlarged budget called for the expansion of these established sports programs and the recruitment of adapted sports instructors (instructors for the disabled people with disabilities) for existing sports facilities [5].

In a study by Sá, Maria Manuel, and four others, the reason that people with disabilities in Portugal did not participate in physical activity and sports was primarily because of a lack of suitable sports facilities. Such people also experience various problems, such as in relation to their financial situation [6]. In the United States, depending on the region, people with disabilities continue to experience various barriers to accessing sports facilities, and according to a survey of 50 sports facilities for people with disabilities in Oregon, major barriers included the entrance, customer service, and shower facilities [7]. Studies that investigated accessibility to most sports facilities for people with disabilities reflected the opinions of people with disabilities in the study [8,9,10].

Unfortunately, according to 2018 survey data on where people with disabilities participate in physical activities and sports, most (61.5%) respondents indicated that they exercised in a park or used a hiking trail near their homes, with 31.8% exercising at home [4]. In other words, although the government is investing heavily in sports centers for people with disabilities, 87.1% of the people with disabilities surveyed indicated that they were not using these facilities, but instead pursuing such activities either at home or in nearby parks.

When the Korean government established a quantitative target for the stimulation of physical activities and sports, local governments made efforts to achieve this target [11]. As a result, the plan has not effectively supported physical activities and sports for people with disabilities based on their needs [12].

For the Korean government’s administration of sports and physical activities for people with disabilities to become effective, it needs to be centered on the concerns of the disabled. This means that the government needs to focus on how to “empower” people with disabilities and create an environment where they can make decisions about their own lives [13]. This is in accordance with the right to self-determination, which is frequently emphasized in the field of sports and physical activity for people with disabilities [14]. In so doing, the Korean government’s supplier-centric administrative system for sports for people with disabilities can be transformed into a consumer-centric system.

Early consumer-centric studies that focused on performance [15,16,17,18,19] reported that a consumer-centric service delivery system not only affects the quality of life of people with disabilities positively but also helps to improve their social participation and empowerment [20,21,22,23,24,25,26,27]. To that end, the needs of people with disabilities should be assessed. Subsequently, based on this assessment, an environment can be provided in which they can easily participate in the sports they choose, increase their physical activity, and enhance their quality of life. Many studies have confirmed that the psychological and physiological health and quality of life of people with disabilities improve through participation in sports and physical activity [28,29,30,31,32].

Disability Rights United Kingdom, the United Kingdom’s leading charitable organization for persons with disabilities, is funded by Sports England and runs a program called Get Yourself Active. This program enhances the general understanding of the benefits of physical activity for disabled persons and provides the environment and opportunities for people with disabilities to participate voluntarily [33]. Similarly, the Netherlands and Canada have improved the quality of life for people with disabilities by providing national support that independently promotes sports, recreation, and physical activity for these individuals [34].

The Korean government has conducted the “Physical Activities and Sports for People with Disabilities Survey” every year since 2006. However, there are only two multiple-choice questions that ask about their needs in the context of facilities for physical activities and sports [3]. Thus, there are no customer data on whether such facilities are required, as the questions do not touch on the diverse and in-depth needs of people with disabilities in terms of the programs and exclude any reference to aspects such the facilities, leaders, or facility management. Thus, to provide effective consumer-centric services, an in-depth analysis of the needs of people with disabilities in Korea in terms of physical activities and sports is necessary. To that end, we use a focus group interview (FGI) approach to gather data to analyze the needs of people with disabilities in Korea in depth.

For qualitative studies, the FGI method, which utilizes an interview guide of open-ended questions, supports a detailed examination of participant experiences, perceptions, opinions, emotions, and knowledge [35]. In addition, by sharing opinions on a topic freely, the participants and interviewers can interact, understanding not only each individual’s thoughts, but also gaining ideas from the group as a whole [36]. Accordingly, many scholars in social science, health science, and evaluation research use FGIs to obtain detailed information that cannot be readily obtained through quantitative surveys [37,38,39,40]. In this study, by using FGIs, we are able to assess the needs of Korean consumers with disabilities in terms of their requirements for participation in physical activities and sports, and identify the underlying environmental issues driving these needs. Ultimately, our objective is to understand the physical activities and sports-related needs of people with disabilities in Korea and how those needs should be reflected in policy and practice to improve their quality of life.

## 2. Materials and Methods

### 2.1. Participants

The purpose of the FGIs was to collect qualitative data from people who had experienced “a specific situation” consistent with our objective [41]. The groups comprised 35 people with disabilities who had participated in physical activities and sports in Korea. When recruiting focus groups in social science research, a mixed group is more effective than a homogeneous group to obtain various viewpoints and experiences [42]. In similar studies, participants in FGIs represented various ages, environments, and disabilities and included subjects with various experiences in physical education to cover both negative and positive experiences, as well as physical activity and quality of life [43,44,45].

In our study, to obtain abundant data in the recruitment process, we considered two factors when screening for people with disabilities who had experience participating in physical education. The first factor was the size of the participant’s city. For the study, we tried to consider needs based on the size of the city by recruiting from both small and large cities in Korea. The second factor was employment status. We deemed that needs would differ based on employment status, since there could be a difference in leisure time between those who were and were not employed.

Regarding the type of disability, in the FGIs, we included a family member who could communicate the opinions of participants with intellectual disabilities, as well as those with physical and visual impairments. The characteristics of the participants are shown in Table 1.

### 2.2. Procedure

To design the focus group guide, we first conducted a literature review on the status of physical activities and sports for people with disabilities in Korea and other countries. The questions for the FGIs were based on what we discovered in this process. Next, we collected data through one-on-one and small-group interviews. These data were coded, and subsequently, through further analysis of the coded data, we derived the participants’ physical activities and sports environment-related needs that could improve their quality of life.

### 2.3. Data Collection

In the first stage of data collection, the current situation was analyzed by collecting domestic and overseas research on physical activities and sports for people with disabilities. The literature review included data between 2018 and 2020 from the “Physical Activities and Sports for People with Disabilities Survey” [3,46,47]—conducted annually in Korea—and data from the United Kingdom’s “Get Yourself Active” [48] and Germany’s “Survey Among People with Disabilities in Leipzig on Participation in Sport” [49]. A summary of the review is presented in Table 2.

In the second stage, the FGI questions were based on the content of the literature review. The questions were completed and reviewed by two associates with PhDs in sports for disabled persons. In the third stage, 35 people with disabilities who had experience in physical activity and sports were recruited.

We recruited these individuals by reaching out to welfare centers and gyms that could identify people with disabilities who would be willing to participate. Subsequently, via e-mail or telephone, we explained the purpose of the study, the interview method, and the time required. Twelve people agreed to participate via email, and twenty-three people agreed to participate via phone. The interview content was also explained, giving the individuals time to contemplate the topic. In the fourth stage, interviews were conducted with small groups of three or four people.

The interview questions were framed according to the method suggested by Krueger and Casey [35], comprising an opening question, an introductory question, a transition question, a key question, and an ending question. The questions are presented in Appendix A.

At the time of the interviews, general information about the interview was provided, consent was obtained for voluntary participation, and a consent form was completed. The content of the consent form included information about the recording of the interview, the destruction of recorded material after the study was completed, the protection of personal information and confidentiality, the participant’s freedom to stop participating in the study, and the fee for participation in the study. Field notes were made during the interviews, and the one-on-one interviews took, on average, 40 min; the small-group interviews lasted, on average, one hour and 30 min. All interviews were conducted in Korean and subsequently translated into English for the study.

### 2.4. Data Analysis and Research Rigor

We analyzed our data according to the FGI content analysis method suggested by Krueger and Casey [35]. First, to exclude any research bias, a research assistant unaffiliated with the study transcribed the FGI content in the form of verbatim records. Second, the transcribed content was compared with the audio files again, and based on the field notes written during the interview process, we added the results of our observations into the transcripts. Third, to analyze the content of the raw data, data were transcribed into Microsoft^®^ Office Word 2019 (Microsoft Corporation, Redmond, WA, USA) and read repeatedly. Subsequently, to extract the themes, meaningful words were entered into Microsoft^®^ Office Excel 2019 (Microsoft Corporation, Redmond, WA, USA). We classified and then reclassified common characteristics of the themes. By repeating the classification process, we were able to identify and analyze common attributes in the responses in terms of frequency, specificity, emotion, and richness. Finally, we determined the categories by extracting themes and theme clusters through this process [48].

We verified the rigor of the study according to the quality assurance criteria of Sandelowski [50]. First, to check reliability, immediately after each interview, the interview was summarized and explained to confirm that the participant and interviewer were in agreement on what had been posited and discussed. Second, we checked the accuracy of the recordings by collating the transcript and audio files several times. All the interviews were transcribed verbatim to ensure data reliability. In particular, the participants with experience participating in physical activity and sports were asked open-ended questions regarding their experiences to enable us to collect sufficient data to reflect their perspectives accurately. After the interviews, the participants’ actions and reactions were recorded in interview logs. These were transcribed within one week of the interviews to ensure that there were no omissions or distortions of the data. In addition, by applying the pause in judgment approach, we were able to avoid any preconceived notions or prejudice in the interview and analysis processes. To ensure suitability, data collection and analysis continued until theoretical saturation was reached. This meant that we were able to capture all the requirements of people with disabilities in terms of physical activities and the sports environment. The validity of the method suggested by Guba and Lincoln [51] was determined as follows: the results were reviewed by a qualitative analysis expert and two sports doctors for people with disabilities; additionally, one professor in sports for people with disabilities reviewed the content that reflected their feedback. The final results were shared with the 35 participants in writing or by e-mail so that they could review these and provide feedback as to any content that needed to be corrected or deleted. As a result, we were able to confirm the accuracy of our final research results.

## 3. Results

A total of 307 meaningful references were derived from the FGIs. Conversations judged to be outside the purpose of the study were deleted, and only meaningful words that fit the purpose were extracted. As captured in the data, the requirements for improving physical activities and sports participation among people with disabilities were classified into programs, facilities, instructors, and health-related information. The themes and theme clusters are listed in Table 3.

### 3.1. Program Needs

The study participants asked for greater support for the facilities where the programs were held, as well as for greater diversification in the programs available in the sports centers.

#### 3.1.1. Equal Support

Participants requested programs that allowed them to exercise in an environment similar to one for people without disabilities.

“*People without disabilities have many private sports facilities close to home and can exercise alone in parks, but very few private sports facilities offer exercise programs for people with disabilities. However, it is too expensive to use. Even the exercise equipment placed in the park has nothing that wheelchair-bound people can [use].*”

Participant 2

“*A lot of people are exercising while watching YouTube at home these days, but when I look at them, most people with disabilities can’t do them. However, there is no movement channel for people with disabilities, and I thought that the government should operate a movement channel for people with disabilities.*”

Non-participant 8

#### 3.1.2. Availability of Various Programs in Sports Centers

The participants were dissatisfied with the fact that only programs at sports centers for people with disabilities were open to them, because these were too limited.

“*My son is gaining weight and he needs to exercise, but it is difficult to exercise because he hates moving. I like the ball, so I want to teach basketball or soccer, but the sports center doesn’t have a ball sports program for the intellectually disabled, so I can’t exercise.*”

Non-participant 1

“*I have severe disabilities, so I always go to exercise with my parents. When I exercise, my parents just wait.*”

Participant 11

### 3.2. Leader Needs

Participants asked for the professional improvement of sports instructors responsible for physical activities for people with disabilities and the enhancement of instructors’ leadership abilities.

#### 3.2.1. Strengthening Instructor Professionalism

Public sports centers or sports centers for people with disabilities are required to appoint leaders with the correct qualifications, namely, qualified instructors for general physical activities and sports for people with disabilities, as well as for adapted elite sports, universal sports, and rehabilitation sports.

“*Even at the sports center for people with disabilities, there are leaders who do not have qualifications as a sports coach for people with disabilities. A leader who is not qualified to teach disabled sports is teaching my daughter, and I thought this was wrong. My daughter has a very poor understanding, so I have to teach it little by little, but if I don’t do it after a demonstration, I just neglect it for the duration of the activity.*”

Non-participant 2

“*I learned swimming from someone who was a disabled sports player in the past, and he definitely teaches me well. I knew exactly how much my disability was, so he taught me in a way that was easy to understand. Apparently, leaders of people without disabilities, even if they are qualified, do not have direct experience with disabilities, so I think there is a limit.*”

Participant 3

#### 3.2.2. Improving Instructor Leadership Skills

The participants said that many leaders did not have the basic abilities to teach physical activities and sports to people with disabilities, emphasizing that improvement in this area was urgently needed. Such improvement included understanding disabled persons, human rights education about people with disabilities, continuous training in sports emergencies and response ability, and improvement in instruction methods.

“*I felt like my coach was ignoring me while I was working out. You cannot do this movement because you are severely disabled. Advanced tactics cannot be used. It hurt my heart when I talked about things like that.*”

Participant 6

“*I went to learn to swim. I’m blind, and coach did a demonstration by saying, “Look at me and follow me…” (laughs). “Coach! I’m blind!” I was very confused. When we exercise, we need a lot of tactile guidance, but it was very difficult to understand the movement because he kept explaining it in words.*”

Participant 13

### 3.3. Sports Facility Needs

The participants indicated that existing sports facilities should be expanded and their operations improved.

#### 3.3.1. Expansion of Facilities for People with Disabilities

Respondents indicated that very few sports facilities were convenient for people with disabilities people to use. The discussion focused on the need for a specific sports facility for people with disabilities, a universal sports facility, a public sports facility, a sports facility for each sport (pool, lawn ball court, etc.), and a school sports facility. Additionally, some requested the installation and expansion of sports equipment for disabled people in the local park.

“*There are very few gyms or swimming pools that are wheelchair accessible to people with disabilities. The weight training equipment is placed so closely that it is difficult for a wheelchair to pass through, and the equipment itself is hardly usable by a wheelchair user. The swimming pool is also very difficult to get into in a wheelchair, and there are very few places where the shower or changing rooms are accessible for people with disabilities.*”

Participant 10

“*Even if a non-disabled sports facility is equipped with facilities for people with disabilities, people with disabilities and people without disabilities can exercise together, but it is a pity that there are so few convenient facilities. So, it is difficult to move while exercising at a sports facility for people with disabilities, far from our home. Too much time is wasted.*”

Participant 4

#### 3.3.2. Improvements at Sports Facilities

Some respondents indicated that the needs of people with disabilities could be accommodated quickly, and the environment readily improved in sports facilities. First, they mentioned the simple need for better publicity regarding the available exercise programs. Second, the general feeling was that they should be able to use the facilities for free. Third, other exercisers, together with the facility staff, should be educated on the different types of disabilities. Finally, several referred to the need for a room for health counseling.

“*When I go to the gym to play badminton, the time for people with disabilities to play is set from 3 to 6 pm. It’s only available on weekends for working people. There are many people who play badminton even on weekdays, so I spend more time waiting than playing. So, even if I ask for an increase in time allotment, I don’t think they will increase it, and they say no. In my opinion, I wish there was no separate time for people with disabilities and those without disabilities. There are six courts, so it’s frustrating because even if they suggest that it would be better to leave only one or two of them empty for people with disabilities, they won’t accept it. They don’t even tell you clearly why you shouldn’t do that, so I stopped working out because I felt bad.*”

Non-participant 7

“*I didn’t even know there was a gym for people with disabilities around my house. I wish they could tell you where the sports center for people with disabilities near my house is and what kind of exercise programs there are by phone or text message. Blind people like us can exercise only when they receive information via text message or phone calls.*”

Non-participant 15

### 3.4. Health Information

Participants responded that they had difficulty receiving information about their health. People with disabilities in Korea receive little information on how to maintain and improve their health. Such information is difficult to find unless it is personally sought out. In addition, as the average age of people with disabilities increases, the number of disabled people who are unable to obtain information on their own through their mobile phones or via the Internet is increasing.

“*Health information for people without disabilities is easy to find on TV or in the mass media, but we cannot find health information about people with disabilities unless we search for it ourselves. I am old and I can’t even use a computer. So, if someone doesn’t tell you, you don’t even know where and what the facilities are. I would like the community to provide information about sports facilities and programs near the house by text message or mail.*”

Non-participant 13

“*I also want to go to a club and exercise with people with disabilities similar to mine. I want to know if there is a disabled sports club in my neighborhood. If there is, I would like to know what kind of sports clubs there are, and if there are clubs with people similar to my age. They said they didn’t know… if I asked the welfare center … If you don’t have a club, you can’t help it, but it may be that you have a club and you are not able to join it. It would be nice if there was a place that would provide such health information.*”

Non-participant 11

The respondents were looking for health information that included content on the importance of exercise programs, as well as information that could motivate them to participate in exercise programs. In addition, they asked for information on the locations of sports facilities, physical fitness measurements, physical activities, and sports programs. They looked to the government as well as the private sector to provide better health information for people with disabilities.

## 4. Discussion

Using FGIs, we were able to analyze in depth the requirements of people with disabilities for their participation in physical activities and sports. As a result, the demands for physical activities and sports of people with disabilities were largely divided into four categories: programs, instructor, facilities, and health information provision. We will focus on these four categories.

Among the first requirements mentioned was equal support for such programs for people with disabilities as well as their establishment within sports centers. There are 1078 gyms and swimming pools in public sports facilities for people without disabilities in Korea. These facilities include programs for weight training, badminton, basketball, volleyball, kendo, table tennis, futsal, golf, children’s physical education, squash, swimming, and aqua aerobics—a very diverse list [52]. Meanwhile, there are just 68 public sports facilities exclusively for people with disabilities, and the programs at these facilities are limited to weight training, swimming, table tennis, badminton, and goalball [53]. These programs do not address the needs of people with disabilities. An environment should be provided that would allow for the self-determination needed to satisfy the three basic psychological needs of autonomy, competence, and enhancement of one’s internal motivation and positive experiences [54,55]. In contrast, Disability Rights United Kingdom provides universal physical activities and sports programs that include both people with and without disabilities, offering many opportunities for people with disabilities to participate in physical activities and sports [48]. Previous studies have shown that when people with disabilities are able to voluntarily participate in these programs, their satisfaction and quality of life increase [24,56]. The Korean and sports organizations for people with disabilities should also reflect these individuals’ needs in order to provide an environment in which people with disabilities can manage their own physical activities and sports.

The second requirement concerned instructors. Respondents mentioned the need for enhancing instructor professionalism and improving leadership abilities. In 2015, Korea established the National Qualification System for Adapted Sports Instructors. However, it is still difficult to employ sports instructors at sports facilities for people with disabilities, as well as at those for people without disabilities [57]. Moreover, in Korea, there is no legal restriction on hiring unqualified sports instructors at sports facilities for people with disabilities [58]. Therefore, instructors without leadership certificates may be teaching people with disabilities. Even instructors licensed to provide sports guidance to people with disabilities are not clear about their roles in such sports facilities and have difficulties due to unstable environments [59]. Similar problems are occurring in other countries as well. The budget for coaches is low, and the job pay is also low [60,61,62]. Therefore, a law should be enacted to only allow the employment of licensed instructors at gyms for people with disabilities, and an environment in which such instructors can work stably should be provided.

There is also the need to improve the working environment for instructors so that they can teach and be leaders [63]. In Japan, for people with disabilities, sports instructors (beginner, intermediate, and advanced), sports coaches, sports trainers, and sports doctors are subdivided, and their roles are clearly defined [64]. In Ireland, its National Disability Authority has a coaching program that supports FGIs among people with disabilities, providing information for physical education teachers, coaches, trainers, managers, and educators. Through such information, adequate sports programs can be developed and provided [43]. In addition, a program to develop the leadership capabilities of instructors should also be provided. Many scholars state that instructors should practice self-reflection on guidance. For this, an opportunity should be provided for instructors to learn how to reflect on their coaching through workshops [65,66,67]. Additionally, coaching networks for leaders should be created and nurtured. It is desirable to provide information on coaching networks to leaders in national sports organizations for people with disabilities [68]. Korea also needs to improve the roles and development of instructors to be able to provide programs at a level that can satisfy people with disabilities.

The third requirement concerned the expansion of sports facilities that could support people with disabilities, along with their improvement. These results also tend to be consistent with previous studies [6,7,8,9,10]. In Korea, facilities where persons with disabilities can exercise are exclusive facilities for people with disabilities or simple sports facilities in local community parks [57]. These facilities are few and far between compared with sports facilities for people without disabilities. This means, for those who want to participate, they have no choice but to travel long distances to utilize such facilities [69]. Research on the causes that prevent persons with disabilities from participating in sports in Korea, as well as potential solutions, is ongoing [70,71]. Previous studies have found that the biggest hindrance to exercise participation among people with disabilities is distance from the exercise facility. In Australia, the perceived barriers to participation in physical activity among children with disabilities also included the lack of transportation and inadequate facilities [72]. Due to such problems, the Netherlands provides travel expenses for people with disabilities to commute to exercise facilities [34]. As a small country, Korea faces difficulties in expanding its facilities. In line with Dutch policy, if people with disabilities could be provided with travel expenses to commute to sports facilities, their participation in physical activities and sports could increase.

Problems related to the operation of sports facilities are often due to a lack of understanding of people with disabilities by the sports facilities’ staff as well as by other exercisers. Human rights issues such as this are continuously emerging in the field of sports for people with disabilities in Korea. Barriers to sports facilities and communication with persons with disabilities not only restrict access but also threaten the quality of participation and render participation unsustainable. Even if individuals participate in physical activity or sports, they have negative stereotypes about the facility operators, which can lead to cessation of participation [73,74]. To promote continued participation in physical activity and sports, it is necessary to focus on whether people with disabilities experience the basic right to use sports facilities [75].

Min and Cho [76] stated that a lack of understanding of people with disabilities’ human rights by the staff at sports facilities for people with disabilities in Korea lowered the satisfaction of people with disabilities who used these facilities. To address these problems, many sports experts for people with disabilities in Korea are working hard to finance the Sports Basic Act, which would give “everyone the right to participate in sports and physical activities and sports without discrimination” [77]. The European Sports Charter states that everyone has the right to participate in sports and that sports must be based on ethics and human dignity [78]. In Australia, the opportunity to participate in physical activity and leisure is recognized as a “right” by law and is considered an essential element contributing to quality of life [79]. As one means of improving facility operations, Korea also needs to enact a Sports Basic Act to legally regulate discrimination against people with disabilities and support their participation at such facilities.

The fourth requirement concerned health information. From one participant who referenced health information, various health information provisions were identified. The health information requested included information that could motivate individuals to participate in sports, as well as more information on the location of sports facilities for people with disabilities. From our interviews, we learned that most people with disabilities do not receive information related to physical activities and sports program availability. Moreover, although they search for health-related information, the information is very limited.

People with disabilities face different barriers to participation in sports and physical activity than people without disabilities [80]. Information about sports and physical activities needs to be better promoted and provided at both national and local levels [28]. In 2014, the English Federation of Disability Sport published a report called “Talk to Me” to encourage people with disabilities to participate in sports. The report set out 10 principles for reaching people with disabilities and offering them sports programs. The first principle was to employ various means of publicity such as social media, local broadcasting, posters, and word of mouth to promote these programs [81]. Ireland has established departments within government ministries and legal institutions to reach out to citizens and ensure equal opportunity for participation in physical activity, as people with disabilities do not have sufficient access to such information. Such actions have created partnerships that raise awareness of the problem, mobilize support, and disseminate information assistance. At the same time, appropriate programs for people with disabilities can be organized and coordinated, primarily within communities and mainstream organizations [43].

In addition, it is very important for health care workers in the community to provide health information and to encourage participation in sports. People with disabilities have a greater reliance on health care workers than the general public [82]. Therefore, health care workers in the community should recommend health information and exercise participation to people with disabilities [83]. In previous research, to create an opportunity to encourage health information and exercise participation, it was necessary to use social capital especially for people with disabilities [84,85,86]. In particular, for people with disabilities, social capital is shared among members through trust, such as social acceptance, life skills, and friendship [85]. In particular, suggestions through social capital for people with disabilities who are participating in sports can promote interaction and participation in exercise [87]. Recently, social capital has been utilized through social networks [83].

In Korea, the government needs to take the initiative to support various means of publicity for such programs, encouraging local communities to pursue these as well so that people with disabilities can obtain health information more readily. If Korea can effectively address the physical activity and sports-related needs of people with disabilities, their quality of life will definitely improve.

## 5. Conclusions

In this study, we used FGIs to determine the needs of people with disabilities in Korea to encourage their participation in physical activities and sports. We found that in terms of programs, more improvement is needed in terms of instructors, facilities, and the provision of health information. Thus far, the approach to physical activities and sports in Korea for people with disabilities has been supplier-centric rather than consumer-centric. Thus, insufficient efforts have been made to listen to the needs of people with disabilities and reflect these in current plans. To improve the quality of life for people with disabilities in Korea, their needs should be reflected in terms of support for their physical activities and engagement in sports.

To provide an opportunity for people with disabilities to choose the exercise program of their choice, it is necessary to research in advance what kind of exercise program people with disabilities want, centering on the local community. To strengthen the professionalism of leaders, it is necessary to provide a stable environment for them in sports facilities and continue training them for capacity building. In terms of facilities, the expansion of sports facilities that can be used by people with disabilities is an urgent priority, and the opinions of users with disabilities must be actively collected and addressed in the operation of these facilities. Additionally, at the national level, information should be continuously provided through mass media and the Internet so that people with disabilities can know the importance of physical activity and sports and manage their own health. To that end, it would be helpful to conduct an in-depth analysis of countries with effective participation policies for people with disabilities and consider how these could be adapted to the situation in Korea.

Some limitations of our study warrant comment and suggest directions for future research. As we analyzed the needs of people with disabilities in small groups using the FGI method, it is difficult to generalize these findings. Thus, based on our results, a larger, quantitative consumer-oriented survey among people with disabilities that could assess their needs in the context of physical activities and sports should be conducted to confirm our results. Additionally, although we identify areas for improvement, further studies should confirm which areas should be improved first.

## Figures and Tables

**Table 1:** Participant characteristics.

Name	Gender	Age	Type	City Size	Job	Name	Gender	Age	Types	City Size	Job
P1	Male	62	CP	Small	No	P18	Male	23	ID	Small	No
P2	Male	45	SCI	Small	Yes	P19	Female	27	ID	Small	No
P3	Female	22	SCI	Small	Yes	P20	Female	63	CP	Small	No
P4	Male	50	MD	Small	No	P21	Male	43	SCI	Small	Yes
P5	Male	26	ID	Large	Yes	P22	Female	48	VD	Large	Yes
P6	Male	24	CP	Large	Yes	P23	Male	47	VD	Large	Yes
P7	Female	60	SCI	Small	No	P24	Male	61	CD	Large	No
P8	Male	36	SCI	Large	Yes	P25	Male	24	CP	Small	Yes
P9	Female	29	CP	Large	Yes	P26	Female	50	VD	Large	No
P10	Male	48	CD	Large	Yes	P27	Female	43	VD	Large	Yes
P11	Male	25	CP	Small	No	P28	Male	59	CD	Large	No
P12	Male	34	CP	Large	No	P29	Male	60	CD	Large	No
P13	Male	35	VD	Large	Yes	P30	Male	66	VD	Large	Yes
P14	Male	30	VD	Large	Yes	P31	Female	31	SCI	Small	Yes
P15	Female	53	SCI	Small	No	P32	Female	54	VD	Small	Yes
P16	Male	56	SCI	Small	No	P33	Male	61	VD	Large	No
P17	Female	48	CD	Large	No	P34	Male	37	SCI	Small	Yes
						P35	Male	21	SCI	Small	Yes

ID: intellectual disability, VD: vision disability, SCI: spinal cord injury, CP: cerebral palsy, CD: cerebrovascular disease, MD: muscular dystrophy.

**Table 2:** Review summary.

Item	Survey on the Participation in Sports for People with Disabilities in Korea		Survey on the Participation in Sports for People with Disabilities in the United Kingdom and Germany
Reference	2018–2020 Physical Activities and Sports for People with Disabilities Survey		United Kingdom: http://www.activityalliance.org.uk/ (accessed on 21 June 2021). Germany: http://www.dguv-forum.de/files/594/09-36-126_DGUV_Forum_9-2009.pdf (accessed on 21 June 2021)
Review Summary	Participation	▪ Sports facilities you want to use near your home: “Parks” in 2018, “Public sports facilities for disabled people” in 2019 and 2020. (However, “Parks” were excluded from the selection in 2019 and 2020.) ▪ Current sports facilities: From 2018 to 2020, the first priority was the park and the second was the home. ▪ Participation type: “Walking and jogging” ranked first in both 2018 and 2020. ▪ Needs for participating in physical education: “Support for participation fee” was required in 2018–2020.	▪ Club-centric: The United Kingdom operates 2711 sports clubs for people with disabilities, centered on 27 sports organizations. In Germany, 1 in 10 people with disabilities join a sports club. ▪ Inclusive facilities and programs: In the United Kingdom, under the “Inclusive Design” policy, Inclusive Fitness Initiative (IFI) sports facilities and sports facilities for people with disabilities coexist together. There are about 400 such facilities across the United Kingdom. ▪ In Germany, when building sports facilities, the regulation of “indoor gym without obstacles” is applied (wheelchair sports, sports for the visually impaired, rehabilitation sports, swimming pools, etc.). ▪ Legal basis: In the United Kingdom, whether the “Inclusive Design” was reflected was determined by how faithfully the Disability Discrimination Act (DDA) obligations were fulfilled. In Germany, according to the German Industrial Standards Association (18040-1), public sports facilities and leisure facilities must be built so that they are not inconvenient.
	Non-participation	▪ Reason for non-participation: Poor health ranked first. ▪ Events you want to participate in in the future: Walking ranked first.	

**Table 3:** Results of environmental needs of physical activities and sports of people with disabilities.

Category	Theme Cluster	Theme
Programs	Equivalent support of the program	Physical activities and sports program for people with disabilities in public and private sports facilities
		Non-face-to-face exercise programs that can be done at home
		An exercise program conducted by a sports club for people with disabilities
		Exercise programs using exercise equipment installed in local parks
	Opening of various programs within the sports center	Physical activities and sports programs according to type of disability
		Physical activities and a sports program for each sport
		Physical activities and a sports program for people with and without disabilities
		Rehabilitation sports programs
		Exercise programs that you can do with your family
		Exercise programs by age group
Facilities	Strengthening the professionalism of instructors	A qualified instructor for physical activities and sports for people with disabilities
		An instructor with professional qualifications in sports
		Sports instructors with disabilities
		Qualified as a disabled sports instructor and as an instructor for those without disabilities
		Instructors with past exercise specialist qualifications
	Improvement of the leadership skills of instructors	Understanding the types of disabilities; education on human rights for persons with disabilities
		Continuous upgrading of skills and knowledge about the sport
		Emergency response ability
		Education in communication methods with people with disabilities (sign language, etc.)
Instructor	Needs to expand facilities available to people with disabilities	Expansion of sports facilities exclusively for people with disabilities
		Expansion of universal sports facilities
		Expansion of sports facilities created for local residents
		Expansion of sports facilities for people with disabilities specializing in sports
		School sports facilities for people with disabilities
		Installation and expansion of convenience facilities for people with disabilities in private sports facilities
		Installation and expansion of sports equipment and facilities in community parks
	Improvement of facility operation aspects	Active acceptance of complaints
		Active promotion of facility and program recruitment
		An environment where there are free tickets to sports classes
		Mandatory education on understanding disabilities for employees and users
		Counseling room for health checkups, maintenance, and promotion
Health information provision	The needs of the entity providing health information	Provision of health information for people with disabilities through mass media at the government level
		Provision of health information by text message or mail at the city hall or community center level of the local community
		Provision of health information by text message or mail to people with disabilities in the local community at the level of the Sports Association for People with Disabilities in the local community
		Provision of health information to disabled users and their families at the welfare center level
		Provision of health information to disabled users and their families at the level of sports facilities
	Content of health information	Information that can motivate exercise by providing information on “disability and exercise,” “necessity of exercise,” “healthy eating habits,”, etc.
		Information on where and what programs are available for people with disabilities in the community
		Locations and related information on sports clubs for people with disabilities
		Related information on physical fitness centers for people with disabilities, etc.

## Data Availability

The data presented in this study are available on request from the author.

## Appendix A

**Table A1:** Semi-structured focus group interview questions.

Question Classification		Question Content	
**Opening question** Brief introduction		Tell us your name? When did you become disabled?	
**Introductory question** Experience participating in physical activities and sports		Do you have experience participating in physical activities and sports?	
		Participant	How did you get involved?
		Non-Participant	Why did not you participate in physical activities and sports?
**Transition question** Difficulty participating in physical activities and sports		Participant	What difficulties did you have in participating in physical activities and sports?
		Non-Participant	Have you ever tried to participate in physical activities and sports?
			What difficulties did you face when trying to participate in physical activities and sports?
**Key question** Needs for physical activities and sports	Programs	Participant	What needs are there for the physical activities and sports program? Please explain in detail.
		Non-Participant	If I participate in physical activities and sports, are there any needs for the program? Please explain in detail.
	Facilities	Participant	What needs do you have for physical activities and sports facilities? Please explain in detail.
		Non-Participant	If I participate in physical activities and sports, are there any needs for the facility? Please explain in detail.
	Instructor	Participant	What needs are there for instructors in physical activities and sports? Please explain in detail.
		Non-Participant	Are there any needs for an instructor if you participate in physical activities and sports? Please explain in detail.
	Health information provision	Participant	What needs do you have for providing health information? Please explain in detail.
		Non-Participant	
**Ending question** Interview impression		Have you ever talked with people around you about the environment of physical activities and sports?	
		Have you tried to change the environment of physical activities and sports yourself?	
		Have you ever felt that your quality of life has increased or decreased through physical activities and sports?	
		How do you feel about the interview?	
